# Self-sampling of the vaginal fluid at home combined with high-risk HPV testing

**DOI:** 10.1038/sj.bjc.6605194

**Published:** 2009-08-04

**Authors:** K Sanner, I Wikström, A Strand, M Lindell, E Wilander

**Affiliations:** 1 Department of Women's and Children's Health, University Hospital, Uppsala, Sweden; 2 Department of Dermato-Venereology, University Hospital, Uppsala, Sweden; 3 Department of Pathology and Cytology, University Hospital, Uppsala, Sweden

**Keywords:** cervix, cytology, screening, HPV test, self-sampling

## Abstract

**Background:**

Around 65% of women with cervical carcinoma in Sweden have not attended an organised screening. We therefore investigated the value of using self-sampling at home in combination with a test for high-risk human papilloma virus (HPV) to increase participation.

**Methods:**

A total of 2829 women 30–58 years old, who had not attended the organised screening for ⩾6 years, were recruited. They were offered self-sampling at home (Qvintip) and recommended to send the collected vaginal fluid to a laboratory for analysis of the presence of high-risk HPV (Hybrid Capture 2 method).

**Results:**

A total of 39.1% of the women accepted home sampling. These women disclosed a relatively high prevalence of high-risk HPV, which decreased with age, from 11.1% in women 30–39 years old to 2.9% in women ⩾50 years . Follow-up disclosed histological cervical intraepithelial neoplasm (CIN) 2–3 lesions in 43.2% of the women with a persistent HPV infection, corresponding to 2.0% of the total number of participating women. The sensitivity of a single smear to detect the histological CIN 2–3 lesions were only 52.6%, even if all abnormal smears (atypical squamous cells of unknown significance (ASCUS)–CIN 3)) were included.

**Conclusion:**

The use of self-sampling at home in combination with testing for high-risk HPV increases the participation rate of the organised screening and detects almost twice as many women with pre-malignant cell alterations (CIN 2–3) in comparison those with a single cytological smear.

A recent nationwide investigation in Sweden disclosed that around 65% of all cervical carcinomas occur in women not attending the organised gynaecological screening and that around 25% are found in women participating in the screening, but with a series of normal cytological specimens preceding the tumour diagnosis. Thus, non-participation and the relatively low sensitivity of cytology are the two major disadvantages associated with the organised screening (Andrae *et al*, 2008). Despite this, the cytological screening has markedly reduced the prevalence of cervical carcinoma by over 50%, with at present <500 women diagnosed in Sweden each year (Bergström *et al*, 1999).

When the organised cytological screening was introduced in the late 1960s, the aetiology of cervical carcinoma was unknown. It is now established that human papilloma virus (HPV) is the major factor for tumour transformation and that DNA of oncogenic (high-risk) HPV types is present in both invasive carcinoma and the pre-malignant mucosal progenitors. As HPV DNA tests are more sensitive than cytology to detect pre-malignant alterations in the cervix, this method is now considered as an adjunct or an alternative to cytology as a preventative screening method (zur Hausen 1991; Meijer *et al*, 2009).

This study was carried out in an attempt to overcome the two most prominent weaknesses of the organised screening, the non-optimal participation rate and the relatively low sensitivity of cytology. Non-participating women were offered the possibility to self-sample the vaginal fluid at home and to send the collected material to a laboratory where a test for high-risk HPV was performed. Women who were HPV positive were admitted to a gynaecological surgery for further evaluation.

## Materials and methods

At the Department of Clinical Cytology, Uppsala University Hospital, all information regarding the organised gynaecological screening is collected in a central database (SymPathy, TietoEnator, Malmö, Sweden). From this data base 3000 women, who were 30–58 years old and had not attended the organised screening for ⩾6 years, were identified. Among the 3000 women selected, 171 had to be excluded because of an incorrect address or a previously performed hysterectomy.

The remaining 2829 women received an information letter and a form by which they could order a device (Qvintip, Aprovix AB, Uppsala, Sweden) to be used for self-sampling of the vaginal fluid at home. The collected material was to be returned in a prepaid envelope to the laboratory, where a test for high-risk HPV was performed. After 3 weeks the women received a letter reminding them to order the self-sampling device. Women who had ordered the device received a letter after about 2 months reminding them to perform the self-sampling of the vaginal fluid. A few women who sent in a sample for analysis more than 3 months after the launch of the study were not included in the investigation.

All participating women received a return letter with the results of the HPV analysis. Women who turned out to be HPV positive were informed that they were going to be called to the Department of Gynaecology for further examination within 3–6 months.

At the gynaecological surgery, women were examined by colposcopy and material was collected for histology, cytology and a repeated HPV test. The histological material was fixed in 10% buffered formalin, embedded in paraffin, sectioned in about 4-*μ* thin sections, which were stained with haematoxylin–eosin stain. The sections were examined in a light microscope and grouped into normal (including cervicitis and metaplasia), cervical intraepithelial neoplasm (CIN) 1 (including condylomas), CIN 2 and CIN 3.

The cytological smears were fixed in 95% ethanol and stained with Pap stain before examination. The smears were grouped into normal, atypical squamous cells of unknown significance (ASCUS), CIN 1, CIN 2 and CIN 3.

The test for high-risk HPV (Hybrid Capture 2, Qiagen AB, Solna, Sweden) identifies 13 high-risk HPV types (16, 18, 31, 33, 35, 39, 45, 51, 52, 56, 58, 59 and 68). The Digene hc2 technique can detect HPV DNA concentrations over 1 pg ml^–1^, which is proportional to the light emission of the positive control and corresponds to 5000 HPV genomes per specimen in the well.

## Results

The self-sampling device was ordered by 1609 women (56.9%) and 1107 (39.1%) performed sampling of the vaginal fluid and sent the material to the laboratory for testing for high-risk HPV. An HPV-positive reaction was obtained in 6.7% (74 out of 1107) of the samples. There was no significant age difference with regard to participation rate. However, the HPV prevalence decreased with age. It was 11.1% in women 30–39 years old and 2.9% in women ⩾50 years (Table 1).

**Figure 1:**
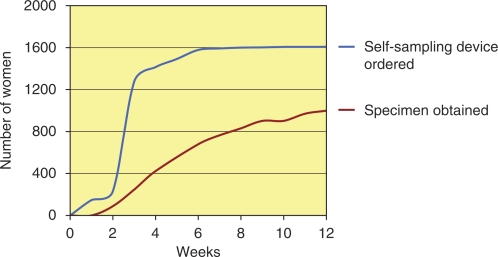
Illustration of the relationship between ordering a self-sampling device and the collection of the vaginal fluid at home with respect to time lapse.

Most women decided to order the self-sampling device within a few weeks and after 8 weeks 1600 women had ordered the device. However, after obtaining the device the decision to collect a vaginal fluid sample was slower, 970 samples were obtained within 12 weeks, and even after that time occasional samples were sent to the laboratory (Figure 1).

The 74 women with a high-risk HPV-positive reaction were admitted to a gynaecological surgery for further examination. However, at that time seven women had moved out of the county and four had chosen to visit a midwife surgery. A repeated test for high-risk HPV, a cervical biopsy and a cytological smear were obtained in 60 of the 63 remaining women. A persistent high-risk HPV infection was seen in 73% (44 out of 60) of the women, 43.2% of which (19 out of 44) showed CIN 2–3 alterations in the cervical biopsies (Table 2). The prevalence of CIN 2–3 in women with persistent HPV infection did not show any marked age variations. It was 40% (8 out of 20) in women ⩾40 years and 45.8% (11 out of 24) in women <40 years.

A biopsy was obtained in 63 of the 74 HPV-positive women and 22 biopsies showed histological CIN 2–3 lesions, corresponding to 2.0% (22 out of 1107) of the total number of women performing self-sampling of vaginal smear. The prevalence of CIN 2–3 was 2.9% among participating women under 40 years and 1.1% among women over 40 years.

The cytological smears taken concomitant with the cervical biopsies at the gynaecological examination showed a normal picture in 75% (45 out of 60) of the cases and various kinds of cell alterations (ASCUS–CIN 3) in 25% (15 out of 60). Among the 19 women with CIN 2–3 in the biopsies, the cytological smear was normal in 47.4% (9 out of 19) of the cases. In only four (21%) of the cytological slides CIN 2–3 cell alterations were recorded (Table 3).

## Discussion

In countries with an organised gynaecological screening, the majority of cervical cancer cases occur among women who have not attended midwife surgeries for collection of a cervical smear (Andrae *et al*, 2008). Thus, to reduce the number of women with cervical cancer, an improvement of the participation rate is an important issue. The offer to non-attending women to self-sample the vaginal fluid at home and send the collected material for testing for high-risk HPV seems to be an attractive method in this respect (Stenvall *et al*, 2006; Wikström *et al*, 2007a, 2007b). The participation rate was unexpectedly high, almost 40% in this study. In a previous pilot study it was even higher, 58% (Wikström *et al*, 2007a, 2007b). This means that a larger proportion of women with an increased risk for development of cervical cancer can be included in the screening programme. It is emphasised that the inclusion of non-attending women in the screening is of special importance because of an increased prevalence of high-risk HPV infections in this category of women (Stenvall *et al*, 2007). An increased incidence of cervical cancer has been reported in this group compared with the participating women (Bos *et al*, 2006; Lindqvist *et al*, 2008).

In addition to the increased coverage obtained by the self-sampling method, its combination with a test for high-risk HPV makes it considerably more sensitive in comparison with a cytological examination. This means that in addition to an increase in coverage, the women are offered an analytical method that is more relevant to identify women with an increased risk to develop cervical cancer as a result of a persistent high-risk HPV infection. That a single cytological examination has a low sensitivity is obvious from this investigation, in which 53% of the women with a biopsy-verified CIN 2–3 lesions on the cervix showed normal cytology. This also explains why around 25% of all cases of cervical cancer occur among women regularly participating in the organised screening (Andrae *et al*, 2008).

A recent meta-analysis has shown that self-sampling and sampling by a doctor has equal validity (Petignat *et al*, 2007). It seems reasonable to argue that self-sampling in combination with high-risk HPV analysis of the collected material is not only an adjunct to sampling of smears, but also a preferable method, as it both increases the participation rate and more accurately identifies women at risk. It is known that the efficiency of cytological screening decreases with age (Gustafsson *et al*, 1995). In some studies the majority of post-menopause women with abnormal smears are HPV negative (Wilander and Wikström, 2008). Further, the prevalence of high-risk HPV infections decreases with age (Wikström *et al*, 2007a, 2007b). For this reason, it now appears that self-sampling, in combination with HPV testing, is considerably more sensitive, and also more specific than cytological screening in middle aged women and older. As HPV tests are becoming cheaper, primary screening in middle aged and older women with HPV testing seems to be favourable in all reasonable respects.

In this study, in agreement with other investigations, it was evident that the prevalence of high-risk HPV decreased with age, from over 11% in women <40 years to <3% in women ⩾50 years. Furthermore, the prevalence was also higher in women performing self-sampling at home in comparison with women participating in the organised cytological screening (Forslund *et al*, 2002). These facts to a large extent explain why the minority of women not participating in the organised screening represents around 65% of all cervical cancer cases (Andrae *et al*, 2008).

It is well known that HPV testing has a higher sensitivity in comparison with cytological examinations (Naucler *et al*, 2007). This fact is obvious in this investigation, in which 47% of the women with biopsy-verified CIN 2–3 lesions presented with a normal cytological smear collected at the same occasion. In addition, it is emphasised that the specificity of the HPV analysis increases with age, concomitant with the decreasing prevalence of persistent HPV infections. This means that at a defined age the HPV test is both more sensitive and more specific than the cytological examinations.

This study shows that 43% of the women with a persistent HPV infection had histological CIN 2–3 lesions on the portio. There was no obvious age difference, although the number of women with CIN 2–3 was slightly higher in women <50 years. In total, 2.0% of all the participating women in the study showed histological CIN 2–3 lesions. In comparison, 0.9% of all cytological smears in the organised gynaecological screening in Sweden show CIN 2–3 and the number of histologically verified CIN 2–3 lesions is about the same (http://ki.se/content/1/c6/05/05/04/Rapport_2006.pdf). This means that around twice as many CIN 2–3 lesions are detected with self-sampling combined with testing for high-risk HPV compared with cytological screening.

In summary, self-sampling at home in combination with a test for high-risk HPV will increase the number of women participating in the organised screening for cervical cancer, and also identify around twice as many CIN 2–3 lesions for treatment with conisation compared with cytological screening.

## Figures and Tables

**Table 1:** Acceptance to perform self-sampling at home and prevalence of high-risk HPV infection in relation to age among 2829 women, 30–58 years old, who had not attended organised cytological screening for over 6 years

**Age (years)**	**Total number of women**	**Number of women performing self-sampling (%)**	**Number of high-risk HPV-positive women (%)**	**Number of women with persistent high-risk HPV infection (%)**	**Number of women with morphological CIN 2–3 lesions (%)**
30–39	984	373 (38)	42 (11.1)	24/33 (73)	11/24 (46)
40–49	968	386 (40)	22 (5.7)	15/18 (83)	7/15 (47)
50–58	877	343 (39)	10 (2.9)	5/9 (56)	1/5 (20)
Total	2829	1107 (39)	74 (6.7)	44/60 (73)	19/44 (43.2)

HPV=human papilloma virus.

**Table 2:** Relationship between a high-risk HPV test and the histological picture in cervical biopsies in 59 women admitted to a gynaecological reception due to a previous positive HPV test

	**HPV test**		
**Histology**	**Positive (%)**	**Negative (%)**	**Total (%)**
Normal	15 (25	9 (15)	24 (40)
CIN 1	10 (16)	7 (12)	17 (28)
CIN 2–3	19 (32)	0 (0)	19 (32)
Total	44 (73)	16 (27)	60 (100)

HPV=human papilloma virus.

**Table 3:** Relationship between cytology and the histological picture in cervical biopsies in 60 women admitted to a gynaecological surgery due to a previous positive HPV test

			**Cytology**		
**Histology**	**Normal**	**ASCUS**	**CIN 1**	**CIN 2–3**	**Total (%)**
Normal	21	1	1	1	24 (40)
CIN 1	15	2	0	0	17 (28)
CIN 2–3	9	4	2	4	19 (32)
Total	45 (75%)	7 (12%)	3 (5%)	5 (8%)	60 (100)

HPV=human papilloma virus.

## References

[bib1] Andrae B, Kemetli L, Sparén P, Silfverdal L, Strander B, Ryd W, Dillner J, Törnberg S (2008) Screening-preventable cervical cancer risks: evidence from a nationwide audit in Sweden. J Natl Cancer Inst 100: 605–606 18445828 10.1093/jnci/djn099

[bib2] Bergström R, Sparén P, Adami HO (1999) Trends in cancer of the cervix uteri in Sweden following cytological screening. Br J Cancer 81: 159–166 10487628 10.1038/sj.bjc.6690666 PMC2374360

[bib3] Bos A, Rebolj M, Habbema JD, van Ballegooijen M (2006) Nonattendance is still the main limitation for the effectiveness of screening for cervical cancer in the Netherlands. Int J Cancer 119: 2372–2375 16858676 10.1002/ijc.22114

[bib4] Forslund O, Antonsson A, Edlund K, van der Brule AJ, Hansson BJ, Meijer CJ, Ryd W, Rylander E, Strand A, Wadell G, Dillner J, Johansson B (2002) Population-based type-specific prevalence of high-risk human papillomavirus infection in middle-aged Swedish women. J Med Virol 67: 535–541 10.1002/jmv.2178 11857534

[bib5] Gustafsson L, Sparén P, Gustafsson M, Pettersson B, Wilander E, Bergstrom R, Adami HO (1995) Low efficiency of cytological screening for cancer *in situ* of the cervix in older women. Int J Cancer 63: 804–809 8847138 10.1002/ijc.2910630610

[bib6] http://ki.se/content/1/c6/05/05/04/Rapport_2006.pdf

[bib7] Lindqvist PG, Hellsten C, Rippe A (2008) Screening history of women in Malmö with invasive cervical cancer. Eur J Obstet Gynecol Reprod Biol 137: 77–83 17210219 10.1016/j.ejogrb.2006.12.005

[bib8] Meijer CJ, Berkhof J, Castle PE, Hesselink AT, Franco EL, Ronco G, Arbyn M, Bosch FX, Cuzick J, Dillner J, Heideman DA, Snijders PJ (2009) Guidelines for human papilloma virus DNA test requirements for primary cervical cancer screening in women 30 years and older. Int J Cancer 124(3): 516–520 18973271 10.1002/ijc.24010 PMC2789446

[bib9] Naucler P, Ryd W, Tornberg S, Strand A, Wadell G, Elfgren K, Rådberg T, Strander B, Johansson B, Forslund O, Hansson BG, Rylander E, Dillner J (2007) Human papillomavirus and Papanicolaou tests to screen for cervical cancer. N Engl J Med 357: 1589–1597 17942872 10.1056/NEJMoa073204

[bib10] Petignat P, Faltin DL, Bruchim I, Tramèr MR, Franco EL, Coutlée F (2007) Are self-collected samples comparable to physician-collected cervical specimens for human papillomavirusDNA testing? A systematic review andmeta-analysis. Gynecol Oncol 105(2): 530–535. 17335880 10.1016/j.ygyno.2007.01.023

[bib11] Stenvall H, Wikström I, Wilander E (2006) Human papilloma virus testing of vaginal smear obtained with a novel self-sampling device. Acta Derm Venereol 86(5): 465–467 16955203 10.2340/00015555-0123

[bib12] Stenvall H, Wikström I, Wilander E (2007) High prevalence of oncogenic human papilloma virus in women not attending organized cytological screening. Acta Derm Venereol 87: 243–245 17533491 10.2340/00015555-0205

[bib13] Wikström I, Stenvall H, Wilander E (2007a) Attitudes to self-sampling of vaginal smear for human papilloma virus analysis among women not attending organized cytological screening. Acta Obstet Gynecol Scand 86(6): 720–725 17520406 10.1080/00016340701303747

[bib14] Wikström I, Stenwall H, Wilander E (2007b) Low prevalence of high-risk HPV in older women not attending organized cytological screening: a pilot study. Acta Derm Venereol 87: 554–555 17989903 10.2340/00015555-0326

[bib15] Wilander E, Wikström I (2008) HPV test for quality control of gynecologic cytological tests. Lakartidningen 105(48–49): 3560, 3564 19133586

[bib16] zur Hausen H (1991) Human papilloma viruses in the pathogenesis of anogenital cancer. Virology 184: 9–13 1651607 10.1016/0042-6822(91)90816-t

